# In Memoriam

**DOI:** 10.21307/jofnem-2021-001

**Published:** 2021-02-06

**Authors:** Philip A. Roberts, J. Ole Becker

**Affiliations:** Department of Nematology, University of California, Riverside, CA 92521

**Seymour Dean VanGundy (1931–2020)**

**Figure:**
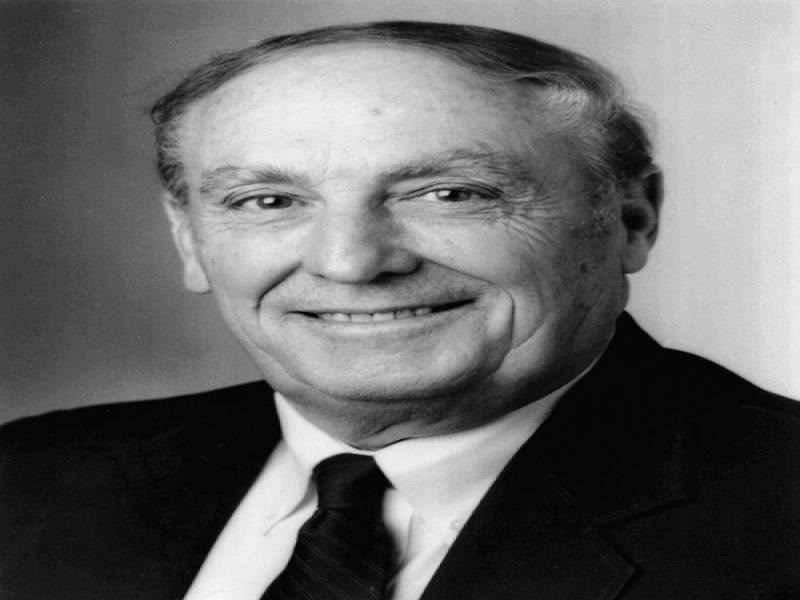


Seymour Dean VanGundy passed away peacefully at home on December 27, 2020. He was born on February 24, 1931, in Toledo, Ohio. Known to family, friends, and colleagues as ‘Van’, he graduated from Monclova High School, Monclova, Ohio, in 1949. Van entered Bowling Green State University on an Edwin Mosley Scholarship and graduated with a B.A. in Biology in 1953. While an undergraduate, he worked part-time at the local H.J. Heinz Crop Research Department, crossing tomatoes and cucumbers and screening cucumbers for cucumber scab resistance under a collaborative program with the Department of Plant Pathology, University of Wisconsin. Plant Pathologist J. C. Walker offered him an assistantship to continue his work on cucumber Angular Leaf Spot when he graduated in 1953.

In 1956, VanGundy finished his PhD research at the University of Wisconsin and continued as a postdoctoral student until February 1957. Dewey J. Raski, Chair of the University of California Statewide Nematology Department, offered him a position in the Nematology Department at UC Riverside. Two months of training with esteemed Nematologist Gerald Thorne prepared Van for his new job before going to California.

He joined the UCR Department of Nematology as a Junior Nematologist in March 1957. At that time, the management and damage control of nematodes attacking citrus in southern and central California was of significant economic interest. In 1958, Van researched and published the first complete life history of the citrus nematode (*Tylenchulus semipenetrans*). Consequently, he worked for many years on the ecology and management of citrus nematodes. Nearly a decade later, he discovered the Sheath nematode (*Hemicycliophora arenaria*), a new species parasitizing desert citrus. With the campus photographer Ken Middleham, they made the first nematode film featuring the feeding and life cycle of the Sheath nematode. In 1964, he was named a Fellow of the American Association for the Advancement of Science (AAAS). Van was appointed Professor of Nematology in July 1968.

During the 1965–1966 academic year, VanGundy spent a sabbatical leave in Australia working with Harry Wallace and Alan Bird to strengthen his interdisciplinary research interest in nematode ecology. In collaboration with UCR colleagues in various departments, Van continued to study resistance in citrus to the citrus nematode, the interaction of multiple nematode associations on citrus and grapevines, and the effects of soil aeration on the ecology of nematodes. He worked with Peter Tsao and Donald E. Munnecke (Dept. Plant Pathology, UCR) on soil fungi/citrus nematode interactions and soil fumigants, respectively. From September 1968 to 1970, he served as Associate Dean for Research in the Graduate Division at UCR.

From 1970-72 Van served as Assistant Vice Chancellor for Research, and from 1972 to 1984, he was Chairman of the Department of Nematology. Through his lobbying efforts, he secured State funding for the first Nematology Quarantine and Isolation facility. In 1977, in collaboration with Diana Wall, he spent a summer at the University of Alaska, Fairbanks, to study nematodes in the black spruce-permafrost ecosystem. In 1979, VanGundy was joint appointed in the UCR Plant Pathology Department and received the title of Professor of Nematology and Plant Pathology. In 1984 he spent a sabbatical leave in Milton Schroth’s laboratory (Plant Pathology, UC Berkeley) studying rhizobacteria. On his return, he served in the College of Natural and Agricultural Sciences as Associate Dean for Research from 1985-88 and Dean of the College from 1988-93.

In the Society of Nematologists, he was instrumental in establishing the Journal of Nematology and served as its first Editor-in-Chief. He served as Vice President and President of the Society of Nematologists and became a Fellow in 1984 and Honorary Member in 1997. In 1978, Van was named a Fellow of the American Phytopathological Society. In 2000, he was appointed to the State Water Quality Control Board- Santa Ana Region by then California Governor Gray Davis and re-appointed in 2006 for a second term by Davis’ successor Governor Arnold Schwarzenegger.

Van retired in 1993, but that did not slow him down, as he continued to work on campus under a five-year re-call agreement and engaged in a range of international program activities for the College and campus. He had a voluntary but active role in the College as Associate Dean for International Programs. He was involved with the University’s extension program, traveling to many countries to stimulate student and visiting scientist exchange with Russia, Vietnam, and Moldova, among others. In 2006, Van was inducted into Moldova’s National Academy of Sciences for his formative role in developing the extension program between UCR and Moldova State University.

Van is survived by his wife of 66 years, Wilma, two children, three grandchildren, and two great-grandchildren. He influenced many people in his life with his kindness and generosity. Van will be deeply missed.

